# IgG-mediated immune suppression in mice is epitope specific except during high epitope density conditions

**DOI:** 10.1038/s41598-018-33087-6

**Published:** 2018-10-16

**Authors:** Hui Xu, Lu Zhang, Birgitta Heyman

**Affiliations:** Department of Medical Biochemistry and Microbiology, Uppsala University, Uppsala, Sweden

## Abstract

Specific IgG antibodies, passively administered together with erythrocytes, suppress antibody responses against the erythrocytes. Although used to prevent alloimmunization in Rhesus (Rh)D-negative women carrying RhD-positive fetuses, the mechanism behind is not understood. In mice, IgG suppresses efficiently in the absence of Fcγ-receptors and complement, suggesting an Fc-independent mechanism. In line with this, suppression is frequently restricted to the epitopes to which IgG binds. However, suppression of responses against epitopes not recognized by IgG has also been observed thus arguing against Fc-independence. Here, we explored the possibility that non-epitope specific suppression can be explained by steric hindrance when the suppressive IgG binds to an epitope present at high density. Mice were transfused with IgG anti-4-hydroxy-3-nitrophenylacetyl (NP) together with NP-conjugated sheep red blood cells (SRBC) with high, intermediate, or low NP-density. Antibody titers and the number of single antibody-forming cells were determined. As a rule, IgG suppressed NP- but not SRBC-specific responses (epitope specific suppression). However, there was one exception: suppression of both IgM anti-SRBC and IgM anti-NP responses occurred when high density SRBC-NP was administered (non-epitope specific suppression). These findings answer a longstanding question in antibody feedback regulation and are compatible with the hypothesis that epitope masking explains IgG-mediated immune suppression.

## Introduction

Passive administration of specific antibodies together with the antigen they recognize can result in dramatic changes in the antibody response as compared to administration of antigen alone (reviewed in^1–3^). This so called antibody feedback regulation can be either positive, resulting in several 100-fold stronger antibody responses, or negative, resulting in more than 99% suppression. The most thoroughly studied feedback regulation is IgG-mediated suppression of antibody responses against erythrocytes. The suppressive ability of IgG has been applied clinically to prevent alloimmunization of RhD-negative women against transplacentally transferred RhD-positive fetal erythrocytes^4–6^. A common experimental approach when trying to elucidate the mechanism behind IgG-mediated immune suppression, has been to immunize mice intravenously with sheep red blood cells (SRBC) or haptenated SRBC^7–11^, or, more recently, with mouse erythrocytes expressing human blood group antigens as transgenes^12–15^. Polyclonal or monoclonal SRBC- or hapten-specific IgG were used as suppressive reagents.

The mechanism behind antibody-mediated immune suppression has been the subject of much speculation since its first discovery in the early 1900’s^16^. Initially, it was postulated that the immune serum “masked” the antigen and prevented it from being recognized by immune cells via so called epitope masking. However, data suggesting that F(ab′)_2_ fragments were much less efficient immunosuppressors than intact IgG^17–21^ prompted the hypotheses that increased clearance of the IgG-antigen complexes via Fc-gamma receptors (FcγRs), or central inhibition of the B cell by co-crosslinking of the B cell receptor (BCR) and the negatively regulating FcγRIIB^22^, were involved. The idea that IgG-mediated immune suppression was Fc-dependent received further support when many laboratories demonstrated that IgG can suppress in a non-epitope specific way: hapten-specific IgG, administered together with haptenated erythrocytes, suppresses the antibody response against erythrocyte epit-opes^10,12,20,21,23,24^, and monoclonal IgG specific for a certain epitope on SRBC suppresses antibody responses also to non-crossreacting epitopes^8,25^. In spite of reports demonstrating that F(ab′)_2_ fragments could suppress^26,27^ and that IgG sometimes suppressed in an epitope-specific way^9,28^, the idea of Fc-dependence dominated. Therefore, the demonstration that IgG efficiently suppressed antibody responses to SRBC in mice lacking activating and/or inhibitory FcγRs^10^ was an unexpected finding and generated some debate at the time^29–31^.

Since then, several reports have confirmed that IgG-mediated immune suppression occurs in the absence of FcγRs^13,15,32,33^ and also in the absence of complement factor C3 (C3), complement factor C1q (C1q), or complement receptors 1 and 2 (CR1/2)^15,33^. These findings suggest that IgG-mediated immune suppression takes place without involvement of the IgG Fc portion and, together with other experimental findings discussed below, suggest that epitope masking is an important explanation for IgG-mediated immune suppression. However, the undisputable existence of non-epitope-specific suppression is apparently in conflict with this idea because it implies dependence of the IgG Fc portion.

Recently, we found that administration to mice of IgG anti-4-hydroxy-3-nitrophenylacetyl (NP), or IgG anti-SRBC, together with SRBC-NP invariably resulted in epitope-specific suppression of the serum IgG response^11^. In a majority of previous studies demonstrating non-epitope specific suppression, the read-out was a direct plaque forming cell (PFC) assay which detects single IgM (but not IgG) anti-SRBC-producing cells within a week after immunization.

We hypothesized that in order for non-epitope specific suppression to occur, two requirements must be fulfilled. First, IgM-responses must be assessed, and, second, the passively administered IgG must bind to an epitope present at high density. In this situation, IgG may be able to prevent B cells from recognizing both the epitopes to which the IgG itself binds (via epitope masking) and neighbouring epitopes (via steric hindrance). The question of Fc-dependence is of utmost importance for understanding the mechanism behind suppression and conflicting data exist. Therefore, we have here analyzed in detail the epitope-specificity of IgG-mediated immune suppression of IgM and IgG serum responses as well as of specific splenic B cell numbers. The results show that IgG always suppresses the response to its specific epitope, regardless of whether IgM or IgG responses are studied and regardless of epitope density. In addition, IgG can also suppress IgM responses to non-specific epitopes, provided that the specific epitopes (to which IgG binds) are present at high density. This suggests that steric hindrance rather than Fc-dependent functions explains non-epitope specific suppression and the data are compatible with the epitope masking hypothesis.

## Results

### IgG anti-NP suppresses IgM anti-NP responses at all NP densities

**Figure 1:**
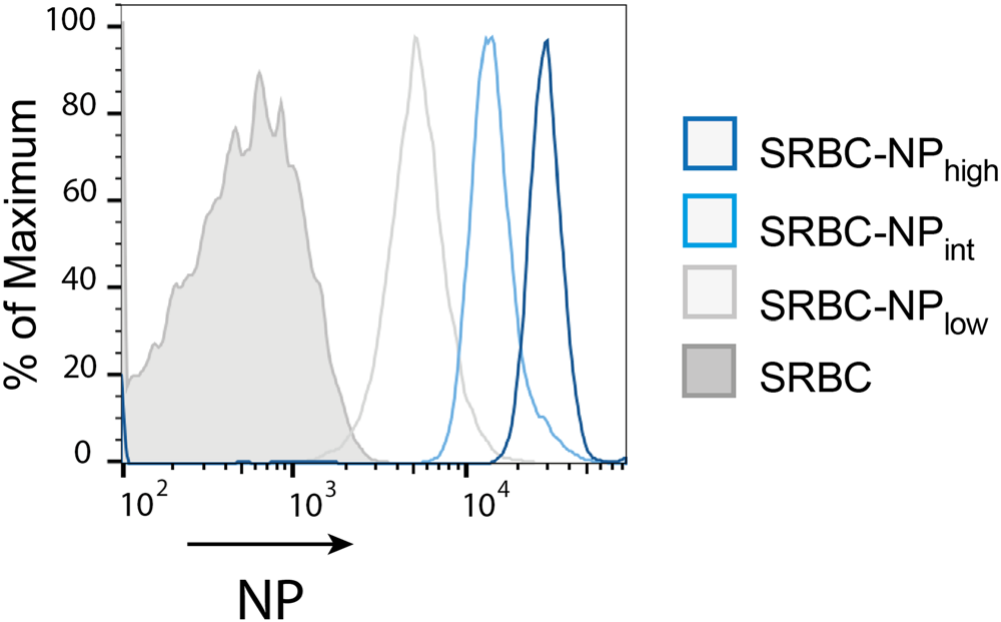
SRBC-NP with high, intermediate, or low density of NP epitopes. High (180 μg/ml), intermediate (60 μg/ml), or low (20 μg/ml) concentrations of NP-ε-aminocaproyl-OSu were incubated with SRBC as described in Materials and Methods. The relative amount of NP conjugated to the erythrocytes was determined by flow cytometry.

**Figure 2:**
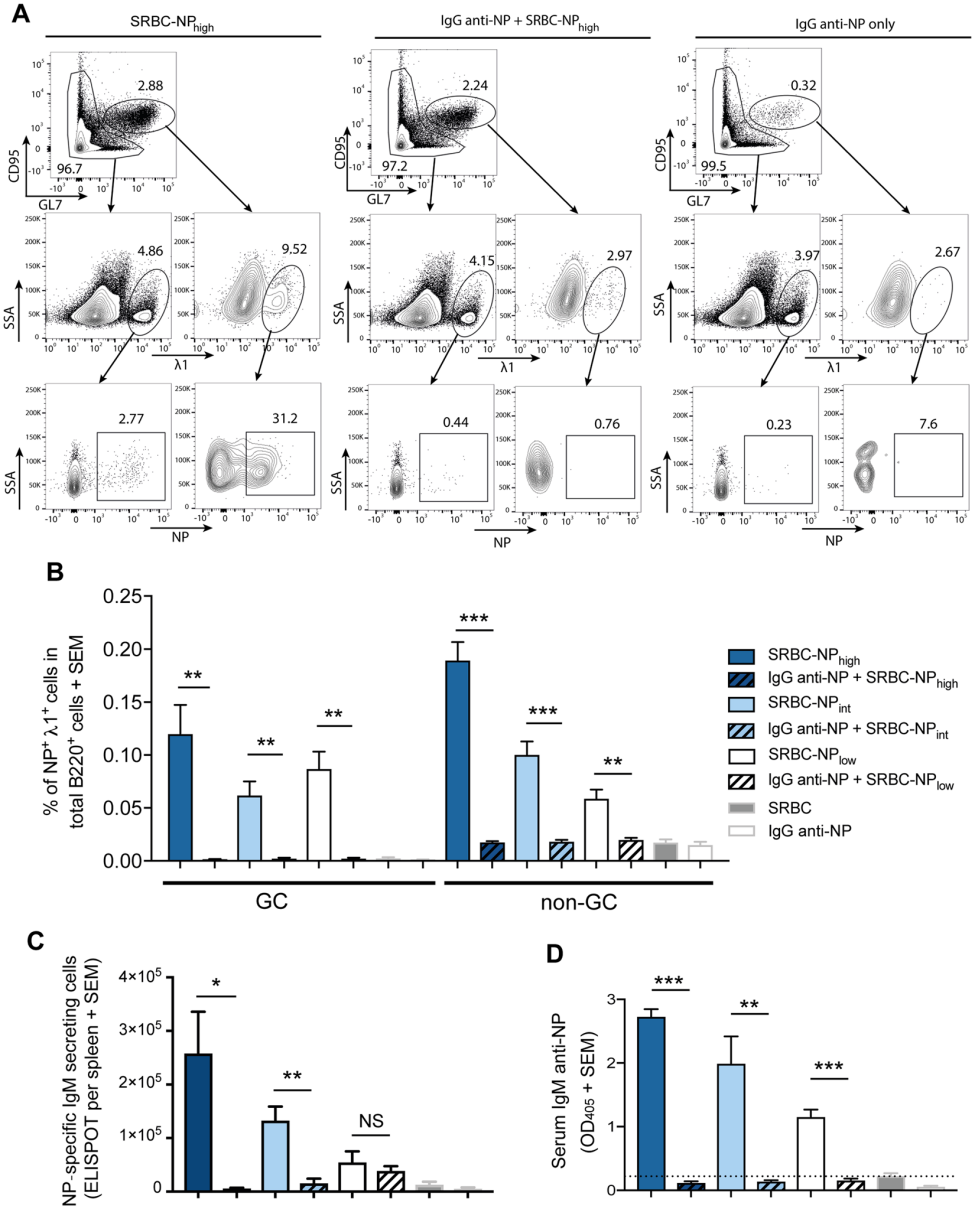
IgG anti-NP suppresses IgM anti-NP responses at all NP densities. C57BL/6 mice were immunized i.v. with 5 × 10^7^ SRBC-NP with high (SRBC-NP_high_), intermediate (SRBC-NP_int_) or low (SRBC-NP_low_) epitope density with or without 30 μg polyclonal IgG anti-NP. Mice immunized with 5 × 10^7^ unconjugated SRBC or with 30 μg IgG-anti NP only were used as controls. NP-specific immune responses were analyzed in spleen and serum samples obtained 5 days after immunization. (**A**) Cells were initially gated for B220^+^ cells. Representative flow cytometry gating for B220^+^ GC (defined as GL7^high^ CD95^high^) and non-GC λ1^+^NP^+^ cells from mice immunized with SRBC-NP_high_ (left), IgG anti-NP + SRBC-NP_high_ (middle) and IgG anti-NP alone (right). (**B**) Frequency of GC and non-GC NP^+^ λ1^+^ cells in total B220^+^ cells. (**C**) NP-specific IgM-secreting cells per spleen. (**D**) Serum IgM anti-NP levels (serum dilution in ELISA = 1:625). The dashed line indicates the mean value of mice immunized with unconjugated SRBC. Representative of four independent experiments with 4–5 mice per group. ns = *p* > 0.05, *p < 0.05, **p < 0.01, ***p < 0.001.

To test the hypothesis that IgG-mediated immune suppression can be either epitope- specific (during low epitope density conditions) or non-epitope specific (during high epitope density conditions), SRBC with high, intermediate, or low density NP were generated (Fig. 1) and mice were immunized with the different SRBC-NP preparations ± polyclonal IgG anti-NP. Five days after immunization, serum and spleen samples were obtained and NP-specific responses were assayed (Fig. 2).

C56BL/6 mice have a genetically restricted antibody response against NP, comprising mainly λ1 light chains and the V186.2 segment of the VHJ558 gene family^34^. Therefore, NP-specific B cells can be identified as B220^+^λ1^+^NP^+^ cells^11^. A representative gating strategy for mice immunized with SRBC-NP_high_, IgG anti-NP + SRBC-NP_high_, or IgG anti-NP alone is shown in Fig. 2A. Cells were first gated as B220^+^ (not shown) and then, using CD95 and GL7 as markers, split into germinal center (GC) B cells (CD95^high^ GL7^high^) and non-GC B cells (top row). The middle row shows GC and non-GC B cells expressing the λ1 light chains and the bottom row λ1^+^ GC and non-GC B cells binding to NP. The results, expressed as % of NP^+^λ1^+^ GC and non-GC B cells of total B220^+^ cells, from one of four experiments is shown in Fig. 2B. In mice immunized with SRBC-NP, GC B cells (GL7^high^ CD95^high^) and non-GC B cells were increased as compared to negative controls immunized with unconjugated SRBC or IgG anti-NP alone (Fig. 2B). In mice transfused with IgG anti-NP together with SRBC-NP, neither the GC- nor the non-GC NP-specific B cell populations increased but remained at the same level as in the negative control mice (Fig. 2B). Thus, IgG anti-NP suppresses the generation of NP-specific GC and non-GC B cells to low, intermediate as well as highly haptenated SRBC-NP.

Next, NP-specific IgM-secreting cells, from the same spleens as above, and serum IgM anti-NP obtained from the same mice, were analyzed. Regardless of NP density, IgG anti-NP suppressed the number of NP-specific IgM-secreting cells (Fig. 2C) and the IgM anti-NP levels in serum (Fig. 2D). Thus, IgG anti-NP administered with either high, intermediate, or low density SRBC-NP consistently suppressed IgM anti-NP responses and the number of NP^+^ splenic B cells.

### IgG anti-NP suppresses IgM anti-SRBC responses at high, but not at low, NP density

**Figure 3:**
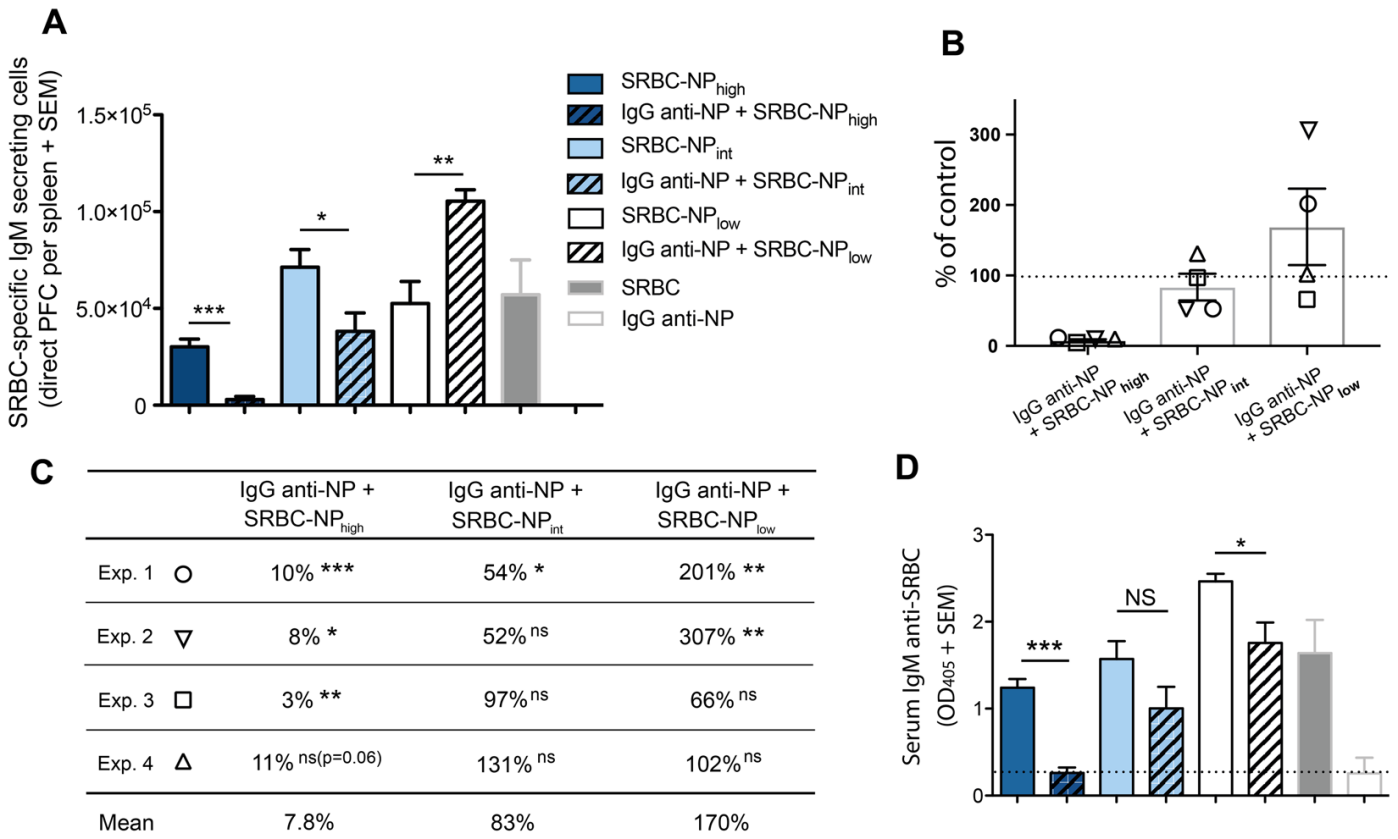
IgG anti-NP suppresses IgM anti-SRBC responses at high, but not at low, epitope density. SRBC-specific IgM responses were analyzed in the mice described in Fig. 2. (**A**) SRBC-specific IgM-secreting cells per spleen (=Exp 1 in **B** and **C**). (**B**,**C**) Summary of the PFC results from all four experiments performed. Suppression is presented as the percentage of the control responses (number of PFCs in mice immunized with SRBC-NP alone, 100%, dashed line) that remains in mice immunized with IgG anti-NP+ respective SRBC-NP. The mean of PFCs in the control groups (representing 100%) were, in the order SRBC-NP_high/int/low_: 30, 080/71, 300/52, 533 (Exp. 1); 24, 793/50, 960/39, 200 (Exp 2); 30, 267/73, 233/136, 027 (Exp. 3); 22, 308/33, 267/63, 967 (Exp 4). *p* values represent comparisons between groups that received IgG anti-NP + SRBC-NP_high/int/low_ and only SRBC-NP_high/int/low_. Each experiment is shown with a different symbol. (**D**) Serum IgM anti-SRBC levels (serum dilution in ELISA = 1:625). The dashed line indicates the mean value of mice immunized with IgG anti-NP alone. ns = *p* > 0.05, *p < 0.05, **p < 0.01, ***p < 0.001.

A crucial question was whether IgG anti-NP, administered together with SRBC-NP, could act in a non-epitope specific manner and suppress also SRBC-specific IgM responses. Splenocytes from the same mice as in Fig. 2 were analyzed for single IgM anti-SRBC-secreting cells in a direct PFC assay. Here, the NP-density on SRBC played a significant role for the outcome of the suppression. In the experiment shown in Fig. 3A (=Exp. 1 in Fig. 3C), IgG caused 90% suppression in mice immunized with SRBC-NP_high_, 46% suppression with SRBC-NP_int_, and no suppression, but a two-fold enhancement, with SRBC-NP_low_. All four experiments performed are summarized in Fig. 3B and C. IgG anti-NP suppressed the PFC response to SRBC-NP_high_ by 89–97%, thus leaving 3–11% of the control response. The response to SRBC-NP_int_ was moderately suppressed or unchanged and the response to SRBC-NP_low_ was unchanged or enhanced.

IgM anti-SRBC in serum from these mice was assessed in ELISA (Fig. 3D). A major decrease was seen in mice transfused with SRBC-NP_high_ together with IgG anti-NP while the response in intermediate or low density groups was only marginally decreased. The IgM anti-SRBC was suppressed by IgG in the high density groups in all four experiments performed. In 5 out of 8 groups immunized with intermediate or low density SRBC-NP, IgG had no effect on the IgM levels. In the remaining 3 groups, the IgM levels were significantly decreased, but invariably the decrease was smaller than in the corresponding high density groups.

A possible explanation for the ability of IgG anti-NP to suppress both IgM anti-NP and IgM anti-SRBC responses at high NP density conditions would be that IgG anti-NP, binding to high density NP-epitopes (but not to low density NP-epitopes), sterically prevents B cells from binding not only to NP- but also to SRBC-epitopes. An indication that this is indeed the case is the fact that IgG anti-NP, incubated with SRBC with different NP-densities, impaired subsequent binding of IgG anti-SRBC to the erythrocytes (Supplementary Fig. 1). The impairment was proportional to the NP density: the higher the NP density the higher the impairment (i.e. the less IgG anti-SRBC could bind). Presumably, this impairment is even more efficient *in vivo* where naive SRBC-specific IgM^+^ B cells, instead of serum IgG, would have to compete with high affinity IgG anti-NP for access to SRBC.

In summary, efficient suppression (>89%) of the IgM anti-SRBC PFC responses occurred with high but not with intermediate or low density SRBC-NP. This agrees with earlier observations of non-epitope specific suppression at high density^24,35^ and with the finding that monoclonal IgG antibodies, recognizing different epitopes on an antigen, have an additive suppressive effect when administered as a blend^14,36^. Blocking experiments *in vitro* directly suggested that this could depend on steric hindrance by the suppressive IgG.

### IgG anti-NP suppresses IgG anti-NP but not IgG anti-SRBC responses at all NP densities

**Figure 4:**
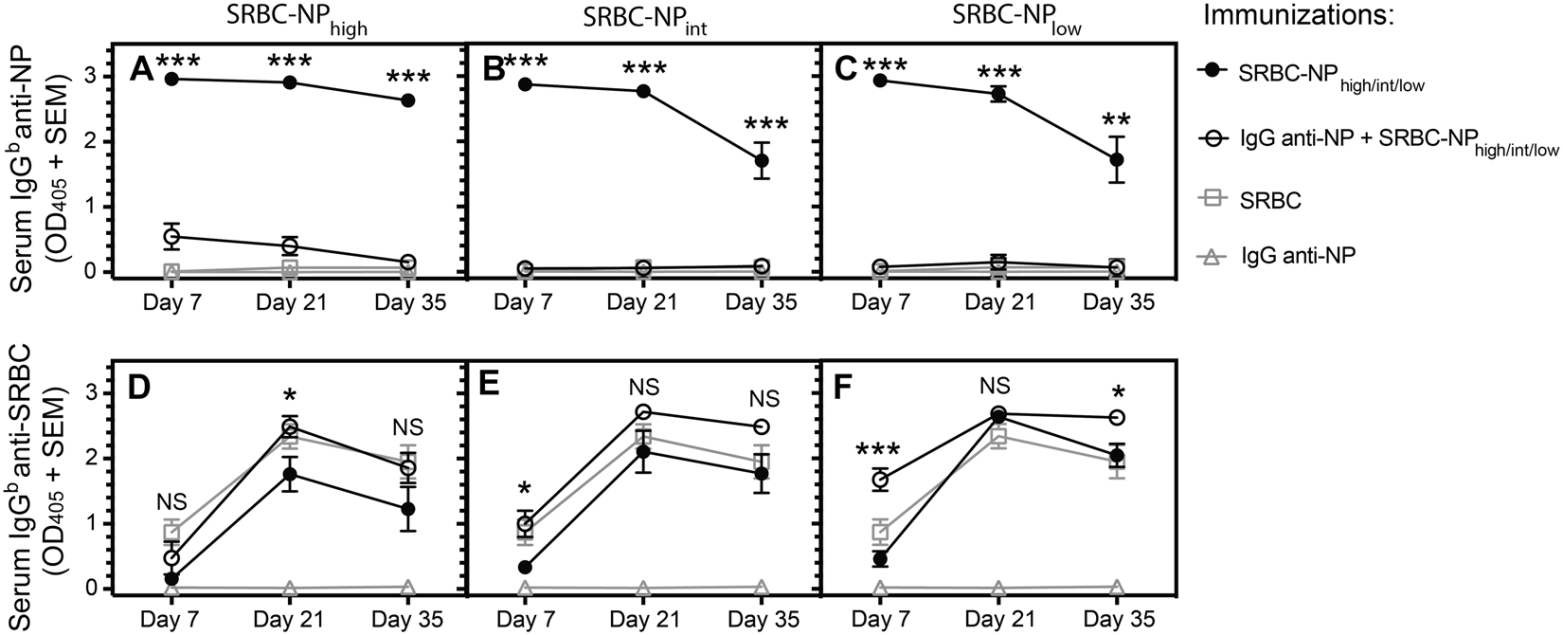
IgG anti-NP suppresses IgG anti-NP but not IgG anti-SRBC responses at all NP densities. C57BL/6 mice (5 per group) were immunized in the same way as described in Fig. 2. Serum samples were collected 7, 21, and 35 days after immunization and assayed for NP- and SRBC-specific IgG^b^ levels (serum dilution in ELISA = 1:625). *p* values represent comparisons between groups that received IgG anti-NP + SRBC-NP_high/int/low_ and only SRBC-NP_high/int/low_. ns = *p* > 0.05, *p < 0.05, **p < 0.01, ***p < 0.001.

IgG anti-SRBC and IgG anti-NP, administered together with SRBC-NP, was previously shown to suppress serum IgG responses in a strictly epitope-specific way^11^. In light of the finding that epitope density was crucial to reveal non-epitope specific suppression of IgM responses (Fig. 3), we here re-addressed the epitope specificity of suppression of IgG responses. The same NP conjugation ratios as in Figs 2 and 3 were used. In analogy with previous observations^11^, IgG anti-NP administered together with SRBC-NP of either of the three epitope densities suppressed the IgG responses against NP but not against SRBC (Fig. 4). Thus, suppression of the IgG response was epitope specific. At a few time points, IgG enhanced the non-epitope specific IgG anti-SRBC response (Fig. 4D–F), thus resembling the effects occasionally seen on the IgM anti-SRBC responses (Fig. 3). Although the overall immunoregulatory role of IgG administered together with erythrocytes is suppressive, enhancement has been noted by a few previous workers^9,37,38^.

## Discussion

**Figure 5:**
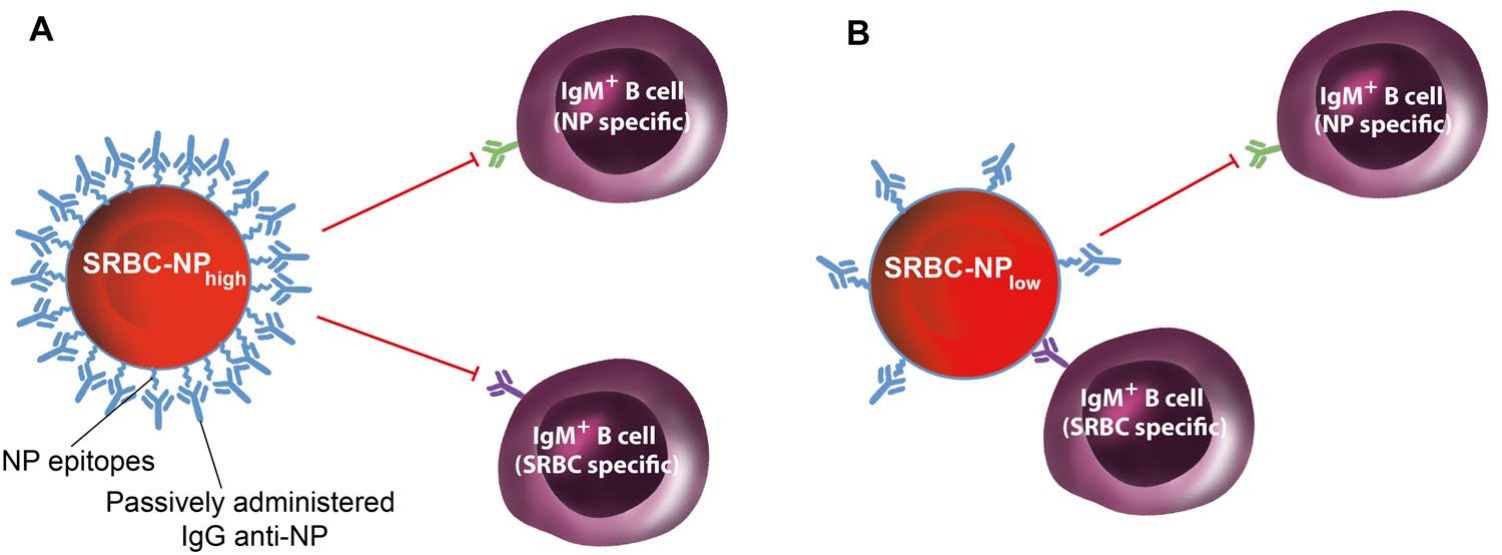
A model for IgG-mediated suppression of IgM responses at high and low epitope densities. (**A**) When NP is present at high density on the SRBC surface, IgG anti-NP will hide both NP epitopes and, due to steric hindrance, SRBC epitopes from recognition by IgM^+^ B cells (which usually have low affinity). This prevents production of both IgM anti-NP and IgM anti-SRBC and will be noted as non-epitope specific suppression. (**B**) When NP is present at low density on the SRBC surface, IgG anti-NP will hide NP, but not SRBC, epitopes from recognition by IgM^+^ B cells. This prevents production of IgM anti-NP but not of IgM anti-SRBC and will be noted as epitope-specific suppression.

The aim of the present study was to find out whether the well documented non-epitope specific suppression by IgG could be reconciled with the equally well documented unperturbed suppression in mice lacking FcγRs. While the former observations suggested Fc-dependence, the latter argued against this. To clarify the specificity of IgG-mediated immune suppression, we analyzed several parameters of B cell responses in the same mice. Our results demonstrate that IgG in most situations suppressed only epitope specific (i. e. NP) responses. However, there was one important exception. When SRBC-NP with high NP-density was administered, IgM anti-SRBC responses, in addition to IgM anti-NP responses, were suppressed by IgG anti-NP. Thus, during these specific conditions, IgG suppressed non-epitope specific (i. e. also SRBC) responses (Fig. 5). Because control mice immunized with SRBC-NP_high_ without IgG responded well to SRBC, it can be excluded that the haptenation completely destroyed SRBC epitopes. Therefore, lack of immunogenic SRBC epitopes cannot explain the suppressed IgM anti-SRBC response in mice immunized with IgG anti-NP + SRBC-NP_high_ shown in Fig. 3. The data presented herein can explain previous, seemingly conflicting, results regarding epitope-specificity of IgG-mediated suppression. They show (i) that both epitope-specific and non-epitope specific suppression of IgM-responses exist, while suppression of IgG-responses is always epitope-specific, and (ii) that epitope density, rather than presence or absence of the IgG Fc portion, determines whether suppression of IgM-responses will be epitope-specific or non-epitope specific. Blocking experiments show that IgG anti-NP, binding to SRBC-NP with different NP densities, is able to impair subsequent binding of IgG anti-SRBC in a density-dependent fashion (Supplementary Fig. 1). This suggests that IgG anti-NP binding to NP-epitopes can sterically hinder also SRBC-specific B cells from binding to SRBC-NP provided the NP-epitopes are present at a high density. Thus, our findings provide an explanation for how IgG can induce either epitope-specific or non-epitope specific suppression and suggest that both are compatible with epitope masking.

An interesting question is why IgG suppresses IgM, but not IgG, responses in a non-epitope specific way during high epitope density conditions. B cells with IgG BCRs develop later in the antibody response and generally have higher affinity than B cells with IgM BCRs. Therefore, IgG^+^ B cells may compete more efficiently than IgM^+^ B cells with the transfused IgG and get access to neighbouring SRBC-epitopes. This would result in an IgG, but not an IgM, anti-SRBC response. Marginal zone B cells are known to respond to thymus-dependent as well as thymus-independent antigens entering the marginal zone from the blood^39,40^ and are presumably responsible for most of the early IgM anti-SRBC responses. A possibility is that intrinsic features of marginal zone B cells render them less efficient than IgG-producing follicular B cells in competing with the transfused IgG anti-NP for SRBC determinants during high epitope density conditions. Yet another possibility would be that erythrocytes are fragmented over time and that small membrane pieces, exposing free SRBC-epitopes, become accessible to SRBC-specific IgG-switched B cells dominating later during the response. Because the NP-epitopes, unlike the SRBC-epitopes, are directly blocked by high affinity IgG anti-NP, neither IgG- nor IgM-producing NP-specific B cells will be able to outcompete the passively administered IgG.

Evidence of epitope-specificity of suppression, in combination with non-epitope specific suppression at high density conditions, is probably the most direct indication of an important role for epitope masking in immunosuppression. Importantly, many other observations are compatible with, although not formally proving, the epitope masking hypothesis. Efficient suppression occurs in the absence of complement and FcγRs^10,13,15,32,33^. Not only intact IgG, but also IgM^41^, IgE^10,42^, and IgG F(ab′)_2_ fragments^10,26,27^ can suppress. Suppression is more efficient with high than with low affinity IgG^20,43^. The suppressive capacity of SRBC-specific monoclonal or polyclonal antibodies correlates with the density of IgG bound to erythrocytes^8,44^ and non-epitope specific suppression requires high hapten density (Fig. 3)^24,35^. Finally, the specific T cell responses are only marginally affected in mice where the antibody response is nearly completely suppressed^10,24^. This is to be expected because SRBC-IgG complexes would be endocytosed and presented as peptides to T cells even when the B cell epitopes are hidden.

On the other hand, some observations are not compatible with the epitope masking model. For example, the inability of IgG F(ab′)_2_ fragments to suppress^17–21^ does not fit with the model. It is not known why some investigators found that F(ab′)_2_ fragments were suppressive^10,26,27^ while others found that they were not^17–21^. One possibility is that F(ab′)_2_ fragments are eliminated faster than intact IgG owing to lack of binding to the protective neonatal Fc-receptor (FcRn)^45^. This may have resulted in concentrations of F(ab′)_2_ fragments too low to induce suppression. Another observation that cannot be reconciled with epitope masking is the failure of IgG to suppress in double knock-out mice lacking C3 and all activating FcγRs (FcγRI, FcγRIII, FcγRIV) but retaining the inhibitory FcγRIIB (suppression worked well in each single knock-out strain)^15^. This finding remains to be clarified since mice lacking C3 generally have severely impaired antibody responses^46^.

Several other mechanisms to explain IgG-mediated immune suppression have been discussed. Central inhibition of B cells via co-crosslinking of BCR and the inhibitory FcγRIIB is hard to reconcile with efficient suppression in mice lacking this receptor^10,13,32,33^. Antigen modulation, i e that IgG after binding to an epitope removes it from the erythrocyte membrane via trogocytosis, is in principle similar to epitope masking and is compatible with many experimental observations. However, non-epitope specific suppression is hard to explain with this model because it would require that IgG anti-NP modulates also SRBC epitopes. This does not seem to happen because SRBC epitopes are still immunogenic in mice immunized with IgG anti-NP and SRBC-NP_high_ (Fig. 4D).

Clearance of erythrocytes from the blood has been suggested to play an important role in IgG-mediated immune suppression, but this hypothesis leaves many experimental findings unexplained. For example, IgG can be administered several days after SRBC (when the erythrocytes are already cleared from the circulation) and still abrogate an on-going antibody response^9,19,32,47^. The most straightforward explanation for this is that the transfused IgG antibodies interfere with the interaction between specific B cells and SRBC, or SRBC fragments, still present in secondary lymphoid organs. This would lead to lack of continued B cell stimulation and result in the reported cessation of antibody production. Other observations not compatible with a major role for clearance are that the ability of IgG to suppress does not always correlate with the ability to induce clearance^11,12,48–50^. Moreover, after administration of IgG anti-SRBC and SRBC, an antibody response to the IgG molecule can be induced although the antibody response to SRBC is efficiently suppressed^51,52^. This would be hard to explain if the IgG-SRBC complexes were removed by clearance but would be expected after epitope masking.

Whether animal models for IgG-mediated immune suppression of erythrocyte responses are relevant for the understanding of how anti-RhD prevents anti-D alloimmunization in humans is a matter of debate^31,48,53^. In spite of the obvious differences, there are also many similarities. Most importantly, antibody-mediated inhibition of antibody responses to intravenous foreign erythrocytes is studied in both situations. The fact that xenogeneic SRBC or allogeneic RhD^+^ erythrocytes are the target antigens may not be as important a difference as postulated. Recent elegant experiments of IgG-mediated suppression in mice immunized with allogeneic erythrocytes (murine RBC expressing transgenic human blood group antigens) arrive at the same conclusions as experiments in mice immunized with SRBC: neither requires FcγRs^10,13,15,32,33^ nor clearance^11,12,48–50^, there is an additive suppressive effect of several monoclonal antibodies recognizing different epitopes^14,36^, and polyclonal IgG preparations are more efficient than monoclonal IgG antibodies^8,14^. In humans there is no strict correlation between clearance and prevention of anti-D alloimmunization^48,54,55^ and, as mentioned above, several studies in mice arrive at the same conclusion^11,12,49,50^. Finally, anti-D administered after transfusion of RhD^+^ erythrocytes^56^ as well as IgG anti-SRBC administered several days after SRBC^9,19,32,47^ effectively inhibit immunization. Attempts to find effective monoclonal or recombinant IgG anti-RhD antibodies to replace the polyclonal anti-D used in the clinic have had limited success^55^. Much of the search has been focused on Fc-related effector functions. Although it will be difficult, or even impossible, to determine exactly how well mouse models reflect the human situation, it may prove fruitful to assume that there is an overlap. This could prompt a search for anti-D antibodies with high affinity and the use of a mixture of antibodies recognizing different epitopes on the RhD antigen.

## Materials and Methods

### Mice

C57BL/6JBomTac mice (C57BL/6) from Taconic Bioscience, Inc (Hudson, NY, USA) were bred and maintained in the animal facilities at the National Veterinary Institute (Uppsala, Sweden). All mice were bred and maintained at the National Veterinary Institute (Uppsala, Sweden) under the supervision of a veterinarian. All experiments were approved by Uppsala Animal Research Ethics Committee and all experiments were performed in accordance with the relevant guidelines and regulations at Uppsala University. Mice were age (6–10 week old mice were used) and sex matched within each experiment.

### Antigens and antibodies used for immunization

Polyclonal IgG anti-NP (allotype a) and IgG anti-SRBC (allotype a) were prepared from hyperimmune BALB/c serum immunized with BSA-NP_20_ in Freund’s complete adjuvant or 10% SRBC i.v. IgG from serum was isolated by affinity chromatography over a Protein-A Sepharose column (Amersham Pharmacia Biotech), dialyzed against PBS and stored at −20 °C. SRBC were purchased from the National Veterinary Institute. For conjugation of SRBC-NP, 4-hydroxy-3-nitrophenylacetic-e-aminocaproyl-OSu (NP-ε-Aminocaproyl-OSu, Biosearch Technologies) was dissolved in dimethylformamide (Sigma-Aldrich) at a concentration of 7.5 mg/ml. Appropriate amounts of this solution was added to 8 ml 2.5% SRBC suspensions in conjugation buffer (0.1 M NaHCO_3_ with 0.15 M NaCl, pH 8.5) to a final concentration of 180, 60, or 20 μg/ml in order to achieve the coupling ratios referred to as SRBC-NP_high_, SRBC-NP_int_ or SRBC-NP_low_. The mixture was incubated for 1 h at room temperature with gentle rotation. Cells were washed 3 times in PBS and stored in PBS up to two days before use.

### Immunization and blood sampling

Mice were immunized with 30 μg NP-specific polyclonal IgG (allotype a) in 200 μl PBS, either alone or followed after 30 min by 5 × 10^7^ SRBC-NP in 200 μl PBS. Other groups were immunized with SRBC-NP or unconjugated SRBC alone. All immunizations were done intravenously in one of the lateral tail veins. Blood samples were collected from the ventral tail artery.

### Enzyme-linked immunosorbent assay (ELISA)

For all ELISAs, 96-well high binding plates (Sarstedt) were used. For the NP-specific ELISA, plates were coated with 100 μl BSA-NP_7_ (50 μg/ml) (Biosearch Technologies) in PBS with 0.05% NaN_3_ at 4 °C overnight. Plates were blocked with 5% dry milk in PBS at 4 °C overnight. For the SRBC-ELISA, 50 μl (25 μg/ml in dH_2_O) poly-L-lysine (Sigma-Aldrich) per well was added for 1 h at 37 °C. After washing twice in PBS, 100 μl 0.25% SRBC in PBS was added to each well and left to settle at room temperature for 1 h. The SRBC were fixed by gently immersing the plates in 0.25% glutaraldehyde in PBS (Sigma-Aldrich) for 10 min. After washing 3 times in PBS, the plates were blocked with 5% dry milk in PBS at 4 °C overnight. Immediately before use, plates were washed three times in PBS and 50 μl of serum samples, serially diluted in dilution buffer (0.05% Tween, 0.25% dry milk and 0.02% NaN_3_ in PBS). Plates were incubated overnight at 4 °C. For detection of IgM, 50 μl alkaline phosphatase-conjugated polyclonal goat anti-mouse IgM (Jackson ImmunoResearch Laboratories), 1:5000 in dilution buffer, was added to each well and incubated over night at 4 °C. The next day, 100 μl of the substrate *p*-nitrophenylphosphate (Sigma-Aldrich) was added and the absorbance at 405 nm was measured after 60 min. For detection of IgG, an allotype-specific ELISA was used to allow distinction between the passively administered IgG^a^ anti-NP and the endogenously produced IgG^b^ anti-NP (this ELISA was also used to detect IgG anti-SRBC). Biotinylated anti-IgG1^b^ (clone B68-2) and anti-IgG2a^b^ (clone 5.7) (both from BD Pharmingen) were mixed 1:1, appropriately diluted in dilution buffer and 50 μl was added to each well. Plates were incubated overnight at 4 °C. After washing, 50 μl alkaline phosphatase-conjugated streptavidin (BD Pharmingen) in dilution buffer was added for 3 h at room temperature. After washing, 100 μl of *p*-nitro-phenylphosphate (Sigma-Aldrich) was added. The absorbance at 405 nm was measured after 60 min. Data was analyzed using SoſtMax soſtware (Molecular Devices).

### Flow cytometry

Polyclonal IgG anti-NP (prepared as described above) was biotinylated using EZ-Link Sulfo-NHS-LC- LC-Biotin (sulfosuccinimidyl-6-biotinamido-6-hexanamido hexaonate) (Thermo Scientific) according to the manufacturer’s instructions. To determine NP density, one million SRBC-NP in 50 μl PBS were stained with 5 μg of biotinylated IgG anti-NP at 4 °C for 30 min. After washing twice in PBS, phycoerythrin-conjugated streptavidin (eBioscience) was added and cells were stained for another 30 min at 4 °C, washed twice in PBS, re-suspended in 300 μl PBS and subjected to flow cytometry. Fifty thousand events were recorded for each sample. To detect NP-specific splenocytes, single cell suspensions in FACS buffer (PBS with 2% fetal bovine serum) were treated with Fc block (anti-CD16/32; BD Biosciences) on ice for 10 min before staining with B220-Alexa700 (clone RA3-6B2), anti-GL7-BV421 (clone GL7), anti-CD95-PEcy7 (clone Jo2), anti-λ1-biotin (clone R11-153) (all from BD Biosciences) at 4 °C for 30 min. After washing, streptavidin-FITC (eBioscience) and NP-PE (Biosearch Technologies) was added. After incubation at 4 °C for 30 min, samples were washed twice in FACS buffer and re-suspended in 300 μl FACS buffer. Subsequently, 2–3 million events were recorded on a LSR Fortessa (BD Biosciences) at the BioVis platform, SciLifeLab, Uppsala, Sweden. Data was analyzed with FlowJo software (Tree Star Inc.).

### Plaque forming cell assay (PFC)

A modified version of the Jerne haemolytic plaque assay^57^ was used. Spleens were harvested 5 days after immunization and single cell suspensions prepared in Hank’s balanced salt solution. One hundred μl of appropriately diluted spleen cells, 25 μl 10% SRBC suspension, and 25 μl guinea pig serum (as a source of complement, diluted 1:10) (National Veterinary Institute) were added to 300 μl 0.5% agarose at 45 °C (50% Seaplaque agarose (low melting point, Amersham) and 50% agarose (United States Biochemical)). The mixture was immediately spread on a microscope slide and incubated in a humid chamber for 3 h at 37 °C. Hank’s balanced salt solution were was used for all dilutions. Triplicate samples were counted “blindly”.

### Enzyme-linked immunospot assay (ELISPOT)

Enzyme-linked immunospot assay (ELISPOT) was used to assess NP-specific IgM-secreting spleen cells. 96-well high binding plates (Sarstedt) were coated with 100 μl BSA-NP_7_ (50 μg/ml) in PBS with 0.05% NaN_3_ and incubated overnight at 4 °C. The plates were washed twice in PBS and blocked with 5% dry milk at 4 °C overnight. After washing three times in PBS, spleen cells were serially diluted in cell culture medium (DMEM with 10% FCS). Hundred μl was added to each well and plates incubated at 37 °C for 18 h. Fifty μl goat anti-mouse IgM-alkaline phosphatase, (Jackson ImmunoResearch Laboratories), diluted 1:1000 in dilution buffer, was added and plates incubated for 3 h at room temperature. After washing three times with PBS, 100 μl of the precipitating substrate 5-bromo-4-chloro-3-indolyl phosphate (Sigma-Aldrich) was added and plates incubated for 1 h at room temperature, washed three times in PBS and dried for 2 h at room temperature. Spots, representing IgM anti-NP producing cells, were counted blindly under a stereo-microscope.

### Statistical analysis

Statistical differences between groups were determined by the two-tailed Student’s t-test. Statistical significance levels were set as: ns, p > 0.05; *p < 0.05; **p < 0.01; ***p < 0.001.

## Electronic supplementary material

Supplementary Information
