# Supplementary material for: IgG-mediated immune suppression in mice is epitope specific except during high epitope density conditions
Source: Sci Rep. 2018 Oct 16;8:15292. doi: 10.1038/s41598-018-33087-6 (PMC6191431; doi:10.1038/s41598-018-33087-6)
Supplement: Supplementary file 1 — Supplementary Information [file 41598_2018_33087_MOESM1_ESM.pdf]

IgG-mediated immune suppression in mice is epitope specific except during high epitope density conditions

Hui Xu[1], Lu Zhang[1], and Birgitta Heyman[1*]

[1]Department of Medical Biochemistry and Microbiology,
Uppsala University, Uppsala, Sweden

*Corresponding author

Supplementary Figure 1. IgG anti-NP, binding to SRBC-NP, impairs subsequent binding of IgG anti-SRBC in an epitope-density-dependent manner.

SRBC were incubated with different concentrations (2 µg/ml, 10 µg/ml, 50 µg/ml, 250 µg/ml) of NP-ε-aminocaproyl-OSu to generate SRBC with different NP-densities. One million unconjugated SRBC or SRBC with different NP densities were incubated with 5 µg IgG-anti-NP-PE at room temperature for 30 minutes. After incubation, cells were washed 3 times with PBS and then incubated with 1 µg IgG-anti-SRBC-APC at room temperature for 30 minutes. Cells were then washed 3 times with PBS and analyzed in flow cytometry. Looking at the y-axis, it can be seen that SRBC-NP_2/IgG anti-NP allowed more binding of IgG anti-SRBC (orange curve) than did for example SRBC-NP_{250} (pink curve). This suggests that IgG anti-NP impairs binding of IgG anti-SRBC to SRBC-epitopes, and that the impairment increases with epitope density. It can be postulated that binding of naive, low-affinity IgM^+ SRBC-specific B cells would be even more impaired than binding of soluble IgG anti-SRBC. Representative of two independent experiments.

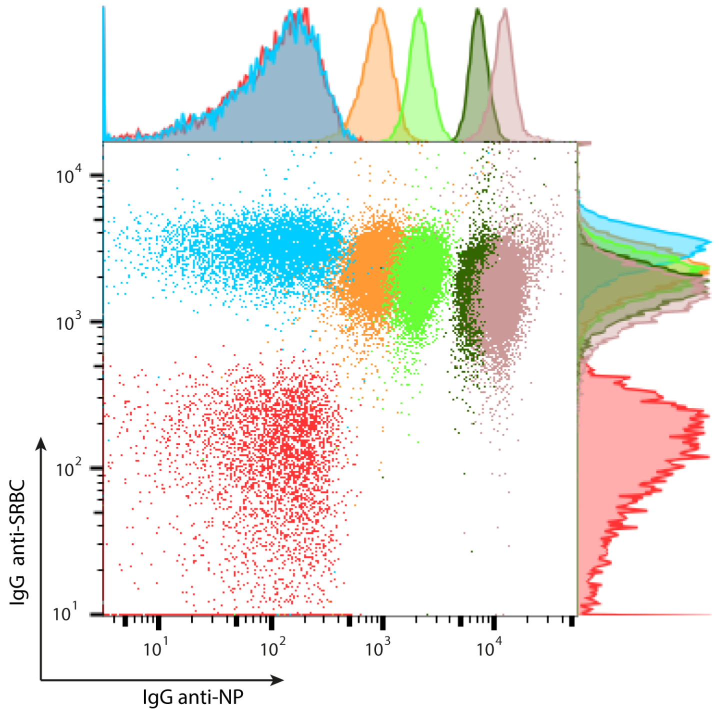

SRBC-NP$_{250}$ + IgG anti-NP + IgG anti-SRBC
SRBC-NP$_{50}$ + IgG anti-NP + IgG anti-SRBC
SRBC-NP$_{10}$ + IgG anti-NP + IgG anti-SRBC
SRBC-NP$_{2}$ + IgG anti-NP + IgG anti-SRBC
SRBC + IgG anti-NP + IgG anti-SRBC
SRBC + Nil (neither IgG anti-NP nor IgG anti-SRBC added)
